# High PVR protein expression marks clear cell renal cell carcinoma with metastatic spread

**DOI:** 10.3389/fimmu.2026.1754848

**Published:** 2026-02-05

**Authors:** Paola Kučan Brlić, Ema Bellulovich, Mijo Golemac, Ante Jakšić, Dean Markić, Karmela Miklić, Suzana Malić, Emina Babarović, Gordana Đorđević, Vesna Šupak Smolčić, Berislav Lisnić, Pini Tsukerman, Guy Cinamon, Stipan Jonjić, Tihana Lenac Roviš

**Affiliations:** 1 Center for Proteomics, Faculty of Medicine, University of Rijeka, Rijeka, Croatia; 2 Department of Urology, Clinical Hospital Center Rijeka, Rijeka, Croatia; 3 Clinical Department of Pathology and Cytology, Clinical Hospital Center Rijeka, Rijeka, Croatia; 4 Clinical Department of Laboratory Diagnostics, Clinical Hospital Center Rijeka, Rijeka, Croatia; 5 Nectin Therapeutics LTD, Jerusalem, Israel

**Keywords:** bladder cancer, metastasis, prognostic marker, PVR, renal cell carcinoma

## Abstract

**Background:**

The poliovirus receptor (PVR) is an emerging therapeutic target currently under clinical investigation in combination with checkpoint inhibitors. Although it is broadly expressed across many malignancies, its prognostic and predictive value in renal and bladder cancers remains underexplored.

**Methods:**

Quantitative immunohistochemical analysis of PVR expression was performed in tumor tissue from patients with bladder cancer or renal cell carcinoma, including clear cell and papillary subtypes. Matched serum samples were analyzed using a custom enzyme-linked immunosorbent assay for total circulating PVR. To distinguish isoforms, we generated an antibody specific to the secreted isoform and developed an isoform-selective assay.

**Results:**

Serum PVR levels were elevated in both bladder and renal cancer patients but did not correlate with tumor tissue expression, whether total or isoform-specific. In bladder cancer, tissue levels correlated with tumor grade and lymphovascular invasion. In renal cancer, expression was subtype-dependent, with only a subset of clear cell carcinoma samples showing tissue positivity. Importantly, reduced overall survival in clear cell carcinoma was linked to PVR expression, and rare cases with high levels consistently progressed to metastatic disease.

**Conclusion:**

In renal tumors, a preliminary subtype-specific pattern emerged: PVR expression was generally low in clear cell carcinoma but enriched in papillary carcinoma. Notably, in the expanded clear cell cohort all cases with high expression were associated with metastatic progression. These findings highlight PVR in tumor biopsies as a potential prognostic marker in clear cell carcinoma and support its further investigation as a biomarker and therapeutic target in renal cancers.

## Introduction

Immune checkpoint inhibitors (ICIs) targeting the PD-1/PD-L1 axis have significantly improved outcomes for a subset of cancer patients (1). However, predicting which patients will benefit from such therapies remains a major clinical challenge (2). Currently, PD-L1 immunohistochemistry (IHC) is the most widely used biomarker, despite well-recognized limitations (3). Eligibility criteria for clinical interventions apply inconsistent positivity thresholds—sometimes as low as 1% PD-L1–positive cells in tumor—and, in some cases, PD-L1 testing is not performed at all prior to treatment due to the biomarker’s limited predictive power.

In this context, the concept of using soluble immune checkpoint biomarkers as non-invasive, systemic, and dynamic indicators has gained attention, though none have yet reached clinical implementation. A meta-analysis on soluble PD-L1 (sPD-L1) as a prognostic marker in ICI-treated patients (4) confirmed that elevated circulating levels correlate with worse prognosis, while also summarizing key sources of inconsistency—such as variation among commercial assay kits, lack of standardized cut-off values, differences between serum and plasma samples, and challenges in distinguishing soluble proteins from extracellular vesicle-bound forms. These limitations have hindered clinical translation.

Despite these shortcomings, PD-L1 expression remains a robust correlate of long-term benefit from ICIs. A recent study (5) demonstrated that tumors from long-term responders exhibited significantly elevated PD-L1 expression. While some PD-L1–negative patients respond to therapy, those with confirmed PD-L1 expression generally show more consistent clinical benefit (6). As the number of available ICIs continues to grow, biomarker-based patient selection is expected to play an increasingly central role in treatment decisions—despite the persistent and puzzling limitations of current biomarkers, which remain incompletely understood.

The poliovirus receptor (PVR, CD155), a molecule functionally related to PD-L1, has emerged as a broadly expressed immune checkpoint ligand associated with poor prognosis (7–9). Also like PD-L1, PVR exists as membrane-bound PVR, which contains a transmembrane domain, and the secreted PVR, which is released extracellularly. PVR engages several immunomodulatory receptors—TIGIT, CD96, KIR2DL5A (inhibitory), and DNAM-1 (activating)—forming a complex axis of immune regulation currently under active clinical investigation. Several ongoing trials are evaluating anti-TIGIT antibodies in combination with other ICIs, underscoring the importance of understanding PVR expression patterns across different malignancies (10). In parallel, NTX-1088—a first-in-class anti-PVR monoclonal antibody—is being evaluated in a Phase I, open-label, multi-center clinical trial (NCT05378425), following encouraging results from preclinical studies.

Both bladder and renal cancer are promising candidates for immune checkpoint inhibitor (ICI) therapy due to their immunologically active tumor microenvironments. Several ICIs are approved for use in these indications. In bladder cancer, PD-1/PD-L1 inhibitors are used as first-line options for cisplatin-ineligible patients and are also approved in second-line and adjuvant settings. In renal cell carcinoma, first-line treatment includes combinations of ICIs or ICIs with anti-angiogenic agents in metastatic disease.

Regarding PVR expression in solid tumors, analysis of TCGA database (11) identified kidney renal papillary cell carcinoma (pRCC) and thyroid carcinoma (THCA) as the two tumor types with the strongest associations between elevated PVR mRNA levels and poor overall survival. While THCA exhibited the highest hazard ratio, pRCC demonstrated greater statistical precision, making it a compelling model for further investigation. Bladder cancer also showed a significant negative prognostic correlation with high PVR expression - in muscle-invasive bladder cancer, PVR is upregulated and associated with aggressive tumor phenotypes and poor prognosis (12, 13).

In this study, we analyzed PVR expression in tumor tissue and matched blood samples from bladder and renal cancer patients. Using a custom-developed ELISA targeting the extracellular domain of PVR, serum PVR levels were elevated in both patient groups compared to healthy donors, while a novel antibody specific for the secreted PVR isoform enabled isoform-specific detection. However, neither secreted isoform reflected tumor tissue expression, consistent with previous observations for PD-L1 (4, 14–16). In the initial cohort of renal tumors, PVR expression was generally low in clear cell RCC (ccRCC) but enriched in papillary RCC (pRCC). Notably, in the expanded cohort, all rare PVR-high ccRCC cases were metastatic, suggesting a potential link to aggressive disease.

## Materials and methods

### Patient samples

Serum and urine samples were collected from 110 patients diagnosed with bladder carcinoma (73 patients; 9 non-papillary, 62 papillary, 2 other) or renal cell carcinoma (37 patients; 25 clear cell carcinoma, 10 papillary, 2 other) who were referred to the Clinical Hospital Centre Rijeka between 2022 and 2023. The median age of the patient diagnosis for renal cancer was 67 years (range: 41–84) and for bladder cancer was 70 years (range: 25–89). All carcinomas were classified according to the WHO and were staged according to tumor-node-metastasis (TNM) system, based on radiologic and pathologic findings. Patients with renal cell carcinoma underwent radical or partial nephrectomy, while those with bladder carcinoma underwent transurethral resection or radical cystectomy. Follow-up for metastatic disease included clinical evaluation, laboratory testing, and radiologic imaging. Demographic, clinical, and laboratory data were collected retrospectively for all patients using an Integrated Hospital Information System (IBIS) and pathological patient database. The control group included serum samples collected from 100 healthy individuals and urine samples collected from 15 patients with no prior history of malignancy. Blood and urine samples were collected from patients at the time of biopsy for pathological confirmation of the disease.

A total of 205 tissue biopsies were included in this study. These included patients from the 2022–2023 serum and urine cohorts (n = 56 bladder cancer and n = 37 renal cancer), as well as bladder cancer patients for whom serum was not collected at the time of biopsy (n = 17, bringing the total number of bladder cases to 73). In addition, tissue-only ccRCC biopsies from patients diagnosed between 2013 and 2015 were included (n = 95). Baseline clinicopathological characteristics of patients are summarized in **Supplementary Tables S1** and **S2**. All samples were retrieved from archived paraffin-embedded material deposited at the Clinical Department of Pathology and Cytology, Clinical Hospital Center Rijeka, Croatia, and the Faculty of Medicine, University of Rijeka. Inclusion criteria included histopathological diagnosis of cancer, availability of formalin-fixed paraffin-embedded tissue specimens, and accessible clinical data obtained from patient medical records.

### Development of ELISAs for detection of soluble PVR

cDNA sequences corresponding to the PVR isoform alpha ectodomain (Transcript ID: ENST00000425690.8) or PVR isoform beta (Transcript ID: ENST00000403059.8), obtained from Ensembl, were subcloned into pcDNA3.1(+)-C-6His vector by Gencript. Plasmid DNA, produced in *E. coli* and purified (NucleoBond Xtra Midi EF kit; Macherey-Nagel), was used for HEK293T cell stable transfection according to standard protocols. The recombinant His-tagged proteins were purified from the cell supernatant using an ÄKTA Pure Liquid Chromatography System (GE Healthcare/Cytiva) and the recombinant proteins were used for immunization and generation of hybridoma producing antibodies. After validation of isoform-specific antibodies, combination of antibodies was used in a sandwich-ELISA. Although we generated a recombinant His-tagged PVR ectodomain and initially established a standard curve, inter-assay variability was markedly reduced by implementing an internal normalization strategy. Specifically, a reference mini-cohort of serum samples was included on every ELISA plate (first 16 positions), which significantly improved reproducibility and internal consistency across experiments, albeit at the cost of an increased number of ELISA plates. All serum samples were tested 3 times, in duplicate. PVR scores were calculated as the sample optical densities (ODs) divided by cut-off values, which were defined as three standard deviations above the mean ELISA ODs of eight healthy donor sera included on each plate.

### TMA preparation and immunohistochemistry

Tissue microarray (TMA) preparation and immunohistochemistry.

The surgery obtained samples were formalin fixed and paraffin embedded. Before immunostaining, HE-stained slides were reviewed by pathologist to select representing tumor sections. TMAs were constructed with MTABooster O1, (Alphelys, Plaisir, France), containing three 1.5-mm core replicates, and used to cut 2.5-μm sections that were then mounted on Microslide SuperFrost Plus slides (WWR, 631-0446) and dried overnight. After blocking with normal goat serum (DAKO, #X0907), anti-PVR antibody (D8A5G, Cell Signaling Technology, #81254) was used at 0.03 µg/mL in SignalStain^®^ Diluent (#8112, Cell Signaling Technology) and incubated for 60 minutes. Endogenous peroxidase activity was blocked with Blocking Solution (Dako, #S2023) for 5 min. Secondary antibody, HRP Rabbit/Mouse ENV (DAKO, #K5007), was incubated for 35 minutes and the IHC visualization was performed with 3, 3’-diaminobenzidine and counterstaining with Mayer’s hematoxylin. Slide scan images were acquired with M8 Microscope & Scanner (PreciPoint GmbH, Germany). PVR IHC intensity was graded weak-moderate-strong and assessed for membrane and cytoplasmic staining for all structures observed. H-score was calculated according following formula: 3 x percentage of cells showing strongly stained membranes + 2 x percentage of cells showing moderately stained membranes + percentage of cells showing weakly stained membranes. The cut-off value for PVR positivity was set to H-score of 10 (13), whereas tumors with H-scores ≥70 were considered to have high PVR expression. For PD-L1, immunostaining was performed using anti-PDL1 antibody (22C3, pharmDx DAKO, #SK006) at 3 µg/mL, incubating for 60 minutes. Immunohistochemical reactivity was assessed using tumor proportion score (TPS) methodology defined as the percentage of viable tumor cells exhibiting membrane PD-L1 staining and quantified from 0% to 100%.

Immunofluorescence HEK293 cells and the transfectant HEK cells expressing either the PVR ectodomain or the secreted form of PVR, were grown on coverslips and processed in two ways. In the first approach, cells were directly incubated with the primary antibody at 4 °C for 30 minutes without prior fixation or permeabilization in methanol. After incubation, coverslips were washed once with 1× PBS, followed by fixation/permeabilization in methanol for 5 minutes. In the second approach, cells were immediately fixed and permeabilized in methanol. In both protocols, after methanol treatment, coverslips were washed twice with PBS, followed by incubation with either both primary and secondary antibodies (for total epitope detection) or only the secondary antibody (for surface epitope detection in the first approach). This allowed assessment of either surface or total epitopes, with an emphasis on intracellular localization. DAPI was added at the end for nuclear visualization. Coverslips were mounted and analyzed using a confocal microscope. Samples were stained with secondary antibodies conjugated to Alexa488 (ThermoFisher, A21121). Cell preparations were imaged using an inverted confocal microscope (Leica DMI8, confocal unit: TCS SP8; Leica Microsystems GmbH, Wetzlar, Germany), equipped with UV (405 nm diode), Ar (488 nm), DPSS (561 nm), and He/Ne (633 nm) lasers. An HC PL APO CS2 (63×/1.40 oil) oil immersion objective was used. Images were acquired using Leica Application Suite (LAS) software (version 3.5.6.21594) in sequential mode. All experiments were performed using the same acquisition settings. Minimal thresholding was applied consistently across all samples to uniformly remove the weakest or emphasize the most intense signals. No gamma correction, pseudocoloring, contrast adjustment, or other image modifications were applied.

### Statistical analysis

Data were collected and statistically analyzed using R 4.5.1 (17–20). Qualitative data were presented as numbers. The Kolmogorov–Smirnov test was applied to assess the normality of distribution. Quantitative data were described using the median and interquartile range (IQR). To compare qualitative variables, such as tumor grades and stages, Fisher’s Exact test was used. For quantitative variables with non-normal distribution, the Mann–Whitney U test was used to compare two groups, while the Kruskal–Wallis test was applied for comparisons involving more than two groups. Survival analysis was performed using the Kaplan–Meier method. Differences in survival between groups were assessed using the log-rank and Gehan–Breslow–Wilcoxon test. A p-value < 0.05 was considered statistically significant.

## Results

**Figure 1:**
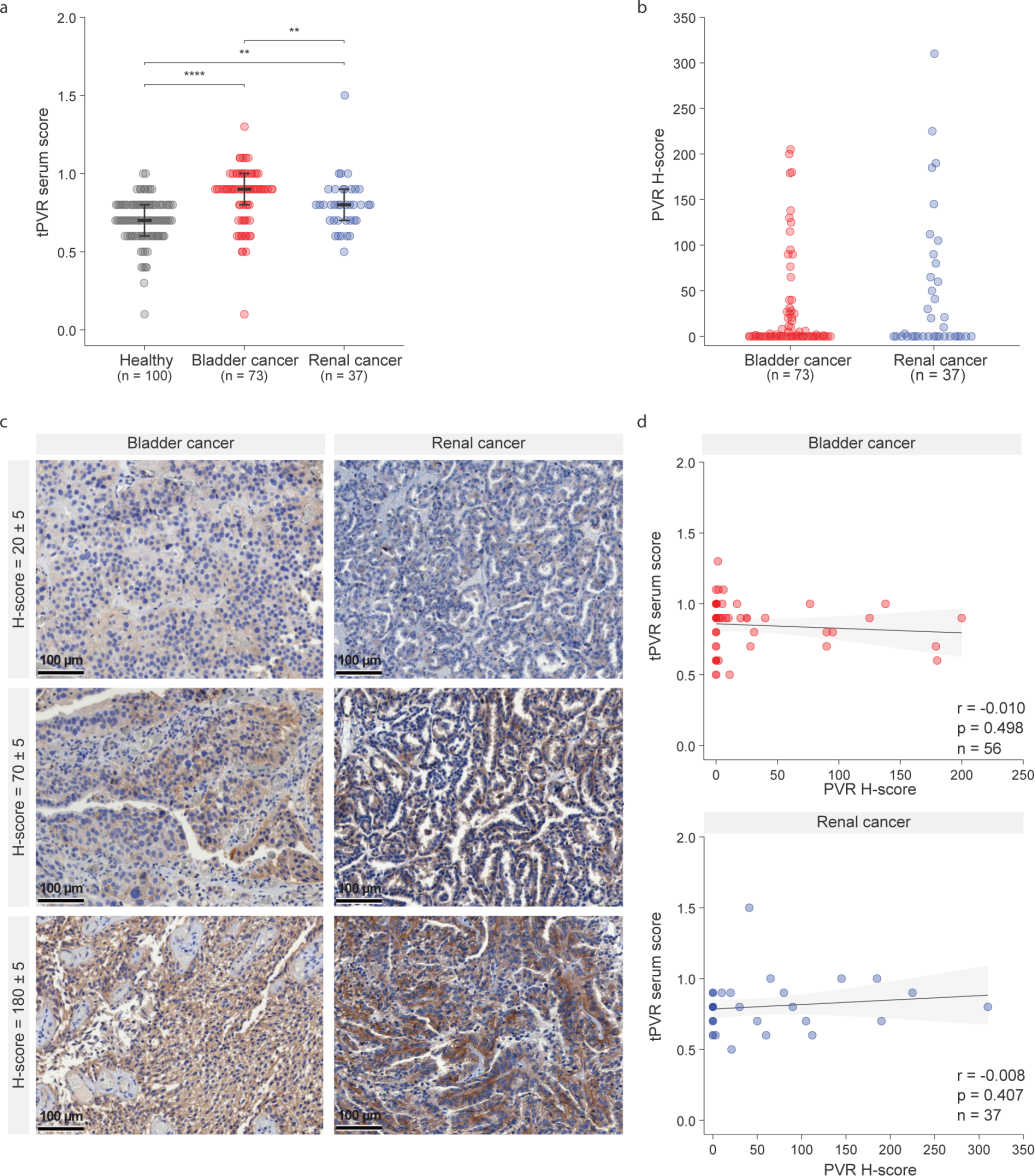
Circulating and intratumoral PVR expression in bladder and renal cancer. **(a)** Serum PVR values measured by a custom-developed ELISA in patients with bladder cancer, renal cancer, and healthy controls. Healthy vs bladder cancer p<0.001, vs renal cancer p= 0.002. Bladder cancer vs renal cancer p=0.008. Statistical analysis was performed using the Kruskal–Wallis test. **(b)** Immunohistochemical (IHC) staining was used to quantify PVR expression in bladder and renal cancer tumor tissues. Quantification was performed using H-scores, which integrate staining intensity and the percentage of positive tumor cells. **(c)** Representative immunohistochemical staining of bladder and renal cancer tissues illustrating varying levels (H-scores) of PVR expression. Scale bars represent 100 µm. **(d)** Correlation analysis between serum PVR concentrations and tumor H-scores in matched bladder (r= -0.010, p= 0.498) and renal cancer (r= -0.008, p= 0.407) samples. Correlation analysis was done using Spearman’s rank correlation coefficient.

Previous studies have reported elevated serum PVR levels in cancer patients compared to healthy individuals (21). To extend these findings to bladder and renal cancers, we developed a custom anti-PVR ELISA and quantified circulating PVR levels in corresponding patient cohorts. As reported for other cancer types, serum PVR levels were significantly higher in both cancer groups compared to healthy controls (healthy vs bladder cancer p<0.001, vs renal cancer p= 0.002; **Figure 1A**). As previously observed, substantial inter-individual variability resulted in considerable overlap between patients and controls, limiting diagnostic precision. These findings suggest that serum PVR may be informative for longitudinal disease monitoring within the same patient, for example decreasing after tumor resection (21). A similar longitudinal relevance has been reported for serum PD-L1, where blood sPD-L1 increased in nearly all patients after anti-PD-1 treatment, but subsequently declined only in responders (22). A second potential application of serum PVR could be the non-invasive identification of patients with exceptionally high expression, paralleling findings for sPD-L1, where high pretreatment levels were linked to poor prognosis or shown to act as an independent prognostic factor (4, 23).

Given the anatomical connection between the urinary tract and the bladder and kidney, we assessed whether PVR could also be detected in urine—an easily accessible sample type. Urinary PVR levels were detectable but generally lower and more variable than those in serum, limiting their reliability for recurrence monitoring (healthy vs bladder cancer p=0.110, vs renal cancer p= 0.670; **Supplementary Figure S1**).

To evaluate whether serum PVR reflects intratumoral PVR expression, we performed immunohistochemical (IHC) analysis on matched tumor samples from the same patients and calculated H-scores for PVR expression. To our knowledge, this is the first study to quantify H-scores in bladder and renal cancer by integrating both the percentage of PVR-positive cells and the intensity of their staining. In both patient cohorts, H-scores identified tumors spanning a range of PVR-expression (**Figures 1B, C**), with representative images for PVR-negative, PVR-positive, and PVR-high tumors, as well as the incidence of PVR positivity using different H-score cut-offs, provided in **Supplementary Figure S2**. However, serum PVR did not correlate with tumor H-scores in either bladder (*r* = –0.010, *p* = 0.498) or renal cancer (*r* = –0.008, *p* = 0.407; **Figure 1D**), consistent with PD-L1 findings (4, 14–16). Correlation between tissue and blood PVR has so far been analyzed only in multiple myeloma (24). As their analysis revealed a link between membrane-localized PVR and soluble PVR levels, we quantified membranous and cytoplasmic expression separately. However, no correlation with serum PVR was observed (bladder cancer: membranous: r=-0.015, p= 0.655, cytoplasmic r= -0.002, p=0.348, renal cancer: membranous r= -0.028, p=0.984, cytoplasmic r=0.024, p=0.177; **Supplementary Figure S3**).

In our analysis, serum PVR levels were generally higher in bladder cancer patients than in those with renal cancer (p=0.008; **Figure 1A**). To explore the molecular basis of this difference, we used UCSC Xena to examine transcript-level expression of PVR isoforms across tumor types (25). Most tumors maintained a stable ratio between the dominant membrane-bound (α) and secreted (β) isoforms compared to their ratio in the corresponding healthy tissue. In contrast, bladder tumors showed a marked shift toward predominant expression of the secreted β-PVR isoform, both in relative proportion and in absolute transcript levels. This observation raised the possibility that serum PVR levels may primarily reflect the secreted isoform and prompted us to selectively quantify secreted PVR while excluding contributions from exosomes, membrane vesicles, apoptotic or necrotic debris, and other membrane fragments.

**Figure 2:**
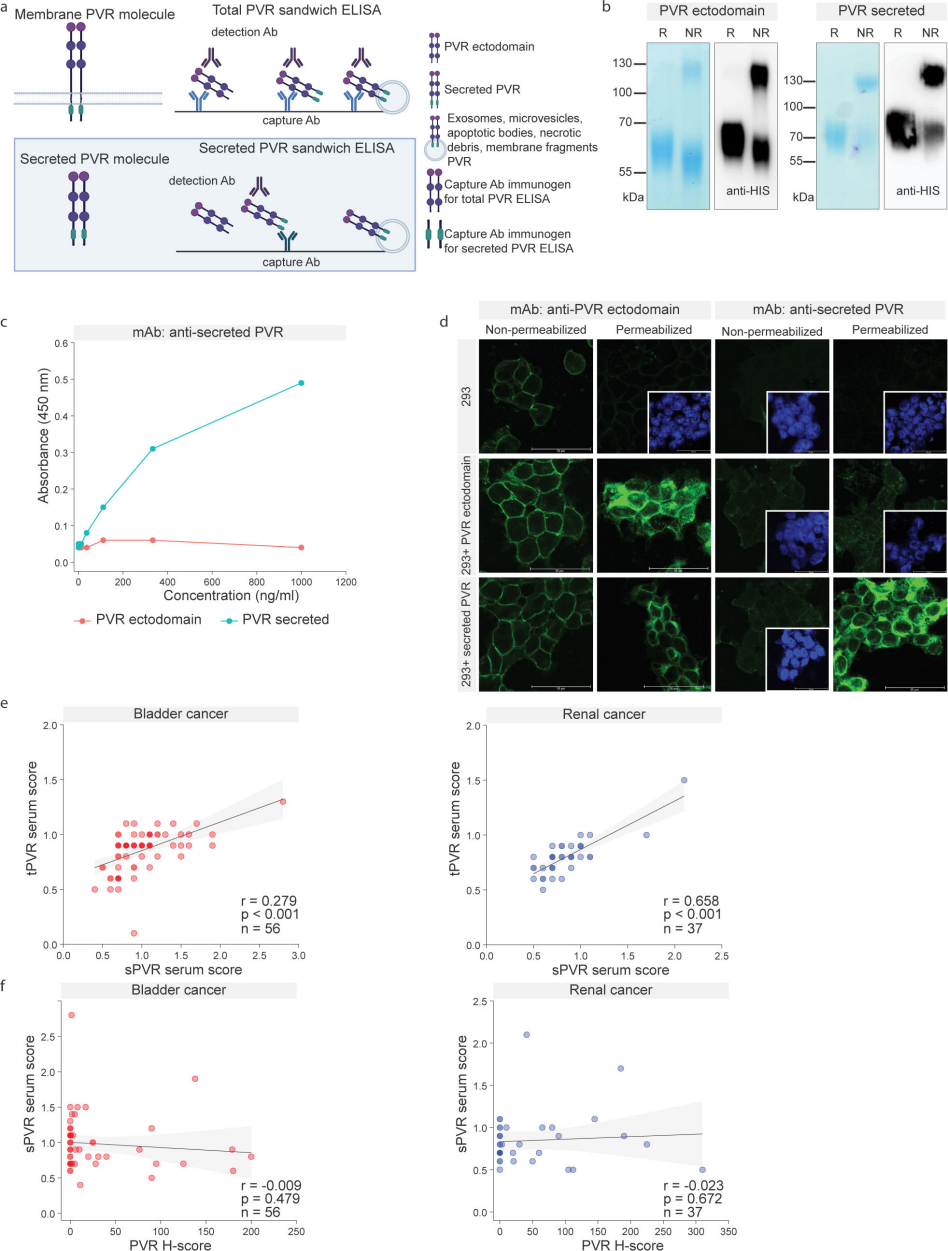
Development and validation of a secreted PVR-specific ELISA. **(a)** Graphical overview of the secreted PVR-specific ELISA, based on an antibody recognizing a peptide unique to the intracellular tail of PVR, which becomes exposed upon secretion. **(b)** Recombinant His-tagged PVR ectodomain and the His-tagged secreted PVR isoform, analyzed by Coomassie staining and anti-His immunoblot under reducing (R) and non-reducing (NR) conditions. **(c)** Validation of the specificity of a monoclonal antibody raised against a peptide located in the intracellular tail of PVR. Clone sPVRβ-KLH.01, coated on an ELISA plate, was probed with either the secreted PVR isoform or the PVR ectodomain. Detection was performed using a commercial polyclonal anti-PVR antibody (STJ28320) and anti-rabbit POD. **(d)** Specificity testing of the monoclonal antibody in HEK293 cells overexpressing either the PVR ectodomain or the secreted isoform. In conditions where no PVR staining (green) was detected, nuclei are shown in blue (DAPI) in the inset frames to confirm the presence of cells. **(e)** Correlation analysis of serum levels measured using the total PVR ELISA and the secreted PVR-specific ELISA across patient samples. Bladder cancer r= 0.279, p<0.001, renal cancer r= 0.658, p<0.001. Correlation analysis was done using Spearman’s rank correlation coefficient. **(f)** Correlation analysis between serum PVR levels (total or secreted) and tumor PVR expression assessed by IHC H-scores. Bladder cancer r= -0.009, p= 0.479, renal cancer r= -0.023, p=0.672. Correlation analysis was done using Spearman’s rank correlation coefficient.

To this end, we developed a second ELISA targeting only the secreted PVR isoform. While the original assay detected the extracellular domain common to both isoforms, the new assay was based on an antibody raised against a peptide unique to the intracellular tail of PVR, which becomes exposed upon secretion (**Figure 2A**). Mice were immunized with this peptide, and both recombinant His-tagged PVR ectodomain and the secreted PVR isoform were produced for antibody validation (**Figure 2B**). Monoclonal antibody (mAb) clone sPVRβKLH.01 was identified as specific for secreted PVR using ELISA coated with sPVRβ-KLH.01 and probed with either the PVR ectodomain or the secreted isoform (**Figure 2C**). Specificity was confirmed in HEK293 cells overexpressing either the PVR ectodomain or the secreted isoform. The sPVRβ-KLH.01 mAb selectively recognized PVR only after membrane permeabilization, indicating intracellular epitope exposure, and only in cells overexpressing the secreted isoform (**Figure 2D**).

The two ELISA assays—total PVR and secreted PVR-specific—showed correlation across serum samples (bladder cancer r= 0.279, p<0.001, renal cancer r= 0.658, p<0.001; **Figure 2E**). Neither assay demonstrated a significant association with PVR expression in tumor tissue (bladder cancer r= -0.009, p= 0.479, renal cancer r= -0.023, p=0.672; **Figure 2F**, **Figure 1D**). These findings are consistent with observations for circulating PD-L1 and support the notion that circulating and tissue PVR levels reflect biologically distinct entities. Consequently, we observed no consistent relationship between blood PVR levels (total or secreted) and either membranous or cytoplasmic PVR expression in tumors, leaving the cellular origin of circulating PVR unresolved.

**Figure 3:**
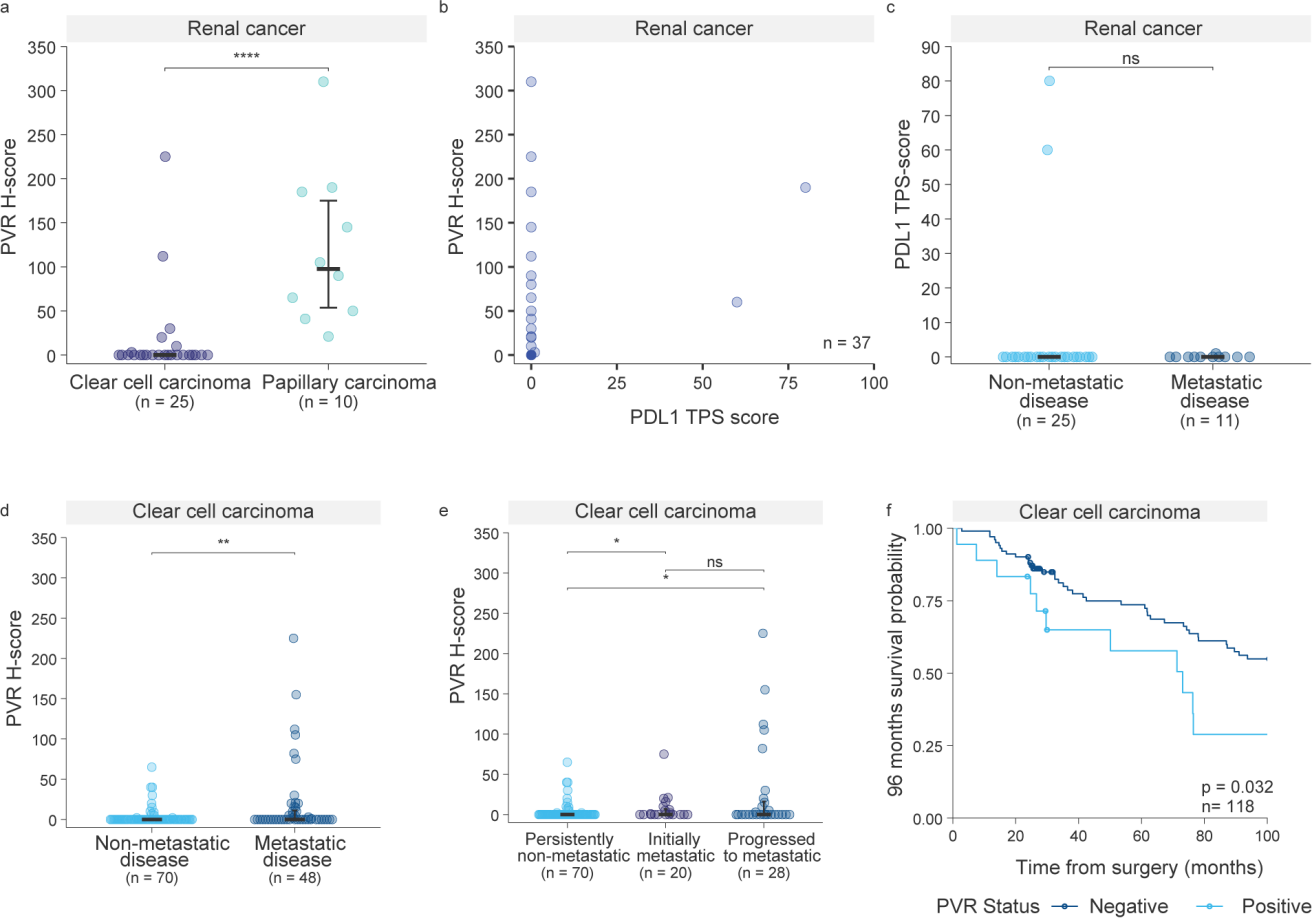
Association of PVR expression with metastatic progression and survival in ccRCC. **(a)** PVR expression across renal cancer subtypes, papillary renal cell carcinoma (pRCC) and clear cell renal cell carcinoma (ccRCC) (p < 0.001). Statistical analysis was performed using the Mann–Whitney U test. **(b)** Two-dimensional plot of PVR H-scores (y-axis) versus PD-L1 TPS scores (x-axis), with each point representing an individual renal cancer patient from the cohort shown in **(a)**. Overlapping points are indicated by correspondingly darker coloring. **(c)** PD-L1 expression stratified by metastatic status (p>0.99) Statistical analysis was performed using the Mann-Whitney U test. **(d)** PVR expression in an expanded ccRCC cohort (n = 118) across metastatic status, comparing metastatic and non-metastatic disease (p = 0.0026). Statistical analysis was performed using the Mann–Whitney U test. **(e)** PVR expression in ccRCC patients stratified by timing of metastasis (persistently non-metastatic vs. initially metastatic, p = 0.010; vs. progressed to metastatic, p = 0.014; initially metastatic vs. progressed to metastatic, p = 0.860). Persistently non-metastatic refers to patients who did not develop metastases until the study endpoint. Initially metastatic includes patients who presented with metastases at the time of diagnosis, while Progressed to metastatic describes those who developed metastatic disease during follow-up. Statistical analysis was performed using the Kruskal–Wallis test. **(f)** Kaplan–Meier analysis of overall survival in ccRCC patients, comparing PVR-positive (H-score ≥10) and PVR-negative (H-score <10) cases. Median survival: 73.09 months for PVR-positive vs. not reached for PVR-negative; log-rank test, p = 0.032; Gehan–Breslow–Wilcoxon test, p = 0.040; HR = 0.489, 95% CI 0.209–1.143.

We next assessed the association between PVR tissue expression and clinicopathological characteristics (**Table 1**). In bladder cancer, higher PVR expression was significantly associated with tumor grade (p= 0.004) and lymphovascular invasion (p= 0.018), consistent with previous studies (12). No such association was detected in renal cancer. Importantly, PVR expression varied markedly across renal cancer subtypes (**Figure 3**). All papillary renal cell carcinoma (pRCC) tumors exhibited PVR levels above the negativity cut-off (H-score = 10 (13),), whereas clear cell renal cell carcinoma (ccRCC) tumors largely remained below this threshold (p<0.001; **Figure 3A**). Notably, the two PVR-positive ccRCC patients showed high tumor PVR expression (H score ≥70), and both patients developed metastases, suggesting a potential link between PVR positivity and aggressive disease in this subtype.

**Table 1:** Association between PVR expression and clinicopathological parameters.

PVR expression									
	Bladder cancer N = 73				Renal cancer N = 37				
Characteristics	Total number (N)	Negative N	Positive N	P value^a^	Characteristics	Total number (N)	Negative N	Positive N	P value^a^
Median age of diagnosis									
<70	33	25	8	p = 0.212	<67	17	11	6	p = 0.509
≥70	40	24	16		≥67	20	10	10	
Sex									
Male	60	39	21	p = 0.525	Male	22	15	7	p = 0.107
Female	13	10	3		Female	15	6	9	
Tumor type									
Papillary	62	43	19	p = 0.458	Papillary	10	0	10	**p < 0.001**
Non-papillary	9	5	4		Clear cell	25	21	4	
Tumor stage									
pTa, pT1	46	34	12	p = 0.127	pT1, pT2	22	11	11	p = 0.500
pT2, pT3, pT4	27	15	12		pT3, pT4	15	10	5	
Tumor grade									
Low grade	26	23	3	**p = 0.004**	Low grade^c^	18	9	9	p = 0.500
High grade	47	26	21		High grade^c^	18	12	6	
LVI									
No LVI	48	37	11	**p = 0.018**	No LVI	7	2	5	p = 0.202
LVI	25	12	13		LVI	30	19	11	

^a^Statistical analysis was performed using Fisher’s exact test. Statistically significant p-values are shown in bold.

As immune checkpoint inhibitors targeting the PD-1/PD-L1 axis are used in renal cancer therapy, and PD-L1 expression is commonly evaluated as a predictive biomarker, we also examined PD-L1 expression in the renal cohort, both overall and in relation to metastasis development. Only a small proportion of tumors expressed PD-L1 compared to PVR, and both patients with high PD-L1 tumor proportion score were also PVR positive (**Figure 3B**). Given the findings in other tumors—where PVR and PD-L1 co-expression has been identified as an independent prognostic factor (26), and PVR alone as an independent prognostic factor (24)—these results suggest a broader applicability of anti-PVR strategies and raise the possibility that certain patients could benefit from combined ICI-based approaches. We further investigated whether high PD-L1 expression was associated with metastasis development; however, neither of the two PD-L1–high patients developed metastases (**Figure 3C**).

Prompted by the potential association between high PVR expression and metastatic disease observed in ccRCC patients, we sought to validate this relationship in a larger cohort. We expanded our ccRCC cohort to include a total of 118 patients by adding cases diagnosed between 2013 and 2015, reasoning that metastatic progression in these cases would now be established. In this larger ccRCC cohort, PVR expression was significantly higher in metastatic disease (p=0.0026; **Figure 3D**).

Importantly, our primary finding was confirmed: although PVR expression is generally low in ccRCC, all cases with high PVR H-scores developed metastases. Stratification by metastasis timing showed the strongest expression in patients who developed metastases later, rather than at diagnosis (**Figure 3E**). This suggests that high PVR expression may predict future metastatic progression and could therefore inform patient monitoring strategies. Although metastases also occur in some patients with lower PVR levels, our data indicate that all ccRCC cases with high PVR expression are at particularly high risk of eventually developing metastases.

Finally, we analysed overall survival (OS) in our ccRCC cohort in relation to PVR positivity, given that PVR has been reported as a poor prognostic factor in several solid malignancies (11, 12, 27–29). Follow-up was truncated at 96 months, a time point beyond which deaths from other causes were shown to exceed RCC-specific mortality in patients (30). Within this timeframe, patients classified as PVR-positive (H-score ≥10) showed significantly reduced survival (median survival: 73.09 months vs. not reached for PVR-negative; log-rank test, p = 0.032; Gehan–Breslow–Wilcoxon test, p = 0.040; HR = 0.489, 95% CI 0.209–1.143) (**Figure 3F**). Analysis of H-score as a continuous variable using Cox regression revealed no significant linear association with OS, consistent with a threshold-dependent rather than dose-dependent effect of PVR expression.

In conclusion, our study highlights the complex biology of PVR, with elevated serum levels in both bladder cancer and renal cancer, independent of tumor tissue expression. Tissue-based analysis revealed subtype-specific PVR patterns, with ccRCC cases showing high intratumoral expression consistently associated with metastasis, suggesting a potentially aggressive phenotype.

## Discussion

Following the success of CTLA-4 and PD-1 inhibitors, anti-PD-L1 antibodies have been approved for advanced urothelial carcinoma and metastatic RCC (30, 31). Maximizing benefit while limiting toxicity remains a major ICI challenge, with only ~12.5% of eligible patients deriving clinical benefit overall (32), while the main stratification tool, PD-L1 IHC, is hampered by technical and interpretive variability (33, 34). Although PD-L1 expression is no longer required for all indications, clinical trials targeting PVR will likely mandate tumor PVR expression for enrollment—paralleling early and recent PD-L1 studies in new cancer types (34–36). Given that PVR is an emerging immune checkpoint molecule implicated in PD-L1 escape pathways and expressed across a broad spectrum of human tumors (10, 37, 38), we assessed its expression in serum and tissue samples. As tissue PD-L1 status does not predict serum levels (4, 14–16), our study shows a similar disconnect for PVR. Due to pronounced inconsistencies in literature and the extreme variability of reported absolute PVR concentrations in blood (21, 39–43), a relative scoring system was applied for serum analyses. The novel splice-specific anti-PVR antibody—enabling detection of alternatively spliced isoform in serum without interference from small membranous or cleaved products, a capability lacking in PD-L1 research—also revealed no correlation with tissue levels. it remains to be determined whether other newly developed isoform-specific antibodies (44) will yield similar results.

In our cohorts, PVR positivity by IHC was observed in approximately 30–40% of tumors using a strict negativity H-score threshold of 10 (13). A recent analysis focusing on muscle-invasive bladder cancer (MIBC) showed broader PVR upregulation (45); however, direct comparison should be made with caution due to differences in cohort composition and methodology. Specifically, bulk mass spectrometry quantifies total protein—including low-level tumor expression and contributions from other cells—whereas IHC reflects clinically defined positivity. Even in ccRCC, a low proportion of PVR-positive cells (H-scores below 10), may still be therapeutically relevant for PVR-targeted strategies, as previously observed for the PD-L1/PD-1. Our NTX1088 antibody—a first-in-class PVR inhibitor—is currently undergoing clinical evaluation in combination with PD-1 ICI. Notably, all pRCC cases in our cohort were PVR-positive, suggesting a potentially targetable population. Papillary RCC is often driven by MET activation (46), and HGF–MET signaling has been shown to regulate PVR levels and promote invasive cellular programs (47). This oncogenic context may underlie the uniform PVR positivity observed in this subtype. However, we refrain from drawing firm conclusions due to the limited number of patients. The relatively small cohort size and single-center design of this study remain important limitations, despite the expanded number of ccRCC cases.

CcRCC is the most common RCC subtype, encompassing both aggressive and indolent clinical courses (48). Our findings indicate that PVR expression is specifically enriched in the more aggressive, metastatic ccRCC cases. This is notable given several unique features of this tumor. First, although tumors with low tumor mutational burden (TMB) are typically unresponsive to ICI, metastatic ccRCC is an exception—responding to ICI despite its low TMB (49, 50). Second, data from the Human Protein Atlas (51) show that DNAM-1, an activating receptor for PVR, has significant prognostic value in one particular cancer type—ccRCC, where high DNAM-1 expression correlates with favorable prognosis. PVR can counteract DNAM-1 expression (52, 53), and RCC can evade immune surveillance through DNAM-1 downregulation (54). Together, these findings suggest a strong rationale for further investigating PVR in metastatic ccRCC, where it may serve not only as a biomarker but also as a driver of immune evasion.

## Conclusions

Our study provides the first integrated analysis of PVR in tissue and matched serum in bladder and renal cancer, using newly developed tools for isoform-specific detection. We confirm that PVR is upregulated in both tissue and circulation, but these two do not correlate. Additionally, we identify a striking tumor subtype-specific pattern, with pRCC showing consistently high PVR expression, while ccRCCs generally exhibited low H-scores—except for a small subset of aggressive, metastatic cases. These findings highlight the potential of tissue PVR as a complementary biomarker in patient stratification and treatment selection, particularly as anti-PVR therapies progress in clinical development.

## Data Availability

The raw data supporting the conclusions of this article will be made available by the authors, without undue reservation.

## Supplementary material

Supplementary Figure 1 Urinary PVR detection in bladder and kidney cancer patients. Urinary PVR concentrations measured by custom ELISA in patients with bladder and renal cancer. Healthy vs bladder cancer p=0.110, vs renal cancer p= 0.670. Statistical analysis was performed using the Kruskal–Wallis test.

Supplementary Figure 2 Incidence of PVR positivity across bladder and renal cancers. Incidence of PVR positivity across bladder and renal cancers, using a cut-off of 10 (A, B, (13)) or 1 (C, D) to define PVR-positive cases. (E) Representative immunohistochemical staining of bladder, papillary and clear cell renal cancer tissues illustrating PVR expression at different H-score thresholds, including the positivity cut-off applied in this study (H-score ≥ 10) and high PVR expression (H-score ≥70). Scale bars represent 100 µm.

Supplementary Figure 3 Correlation analysis between tissue and serum PVR. (A) Membranous and (B) cytoplasmic PVR expression in tumor tissues was quantified separately and compared with serum PVR levels. Bladder cancer: membranous: r=-0.015, p= 0.655, cytoplasmic r= -0.002, p=0.348, renal cancer: membranous r= -0.028, p=0.984, cytoplasmic r=0.024, p=0.177. Correlation analysis was done using Spearman’s rank correlation coefficient.
