# Supplementary material for: High PVR protein expression marks clear cell renal cell carcinoma with metastatic spread
Source: Front Immunol. 2026 Feb 5;17:1754848. doi: 10.3389/fimmu.2026.1754848 (PMC12916676; doi:10.3389/fimmu.2026.1754848)
Supplement: Supplementary Figure 1 — Urinary PVR detection in bladder and kidney cancer patients. Urinary PVR concentrations measured by custom ELISA in patients with bladder and renal cancer. Healthy vs bladder cancer p=0.110, vs renal cancer p= 0.670. Statistical analysis was performed using the Kruskal–Wallis test. [file DataSheet1.pdf]

a

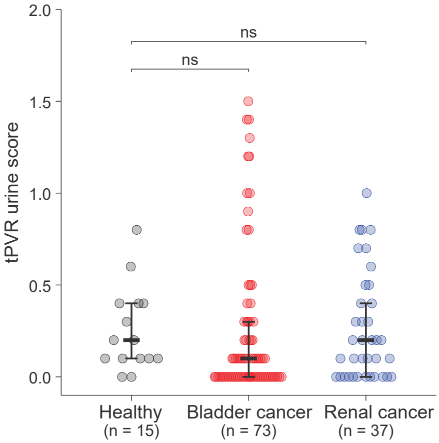

ns
ns
tPVR urine score
2.0
1.5
1.0
0.5
0.0
Healthy
(n = 15)
Bladder cancer
(n = 73)
Renal cancer
(n = 37)

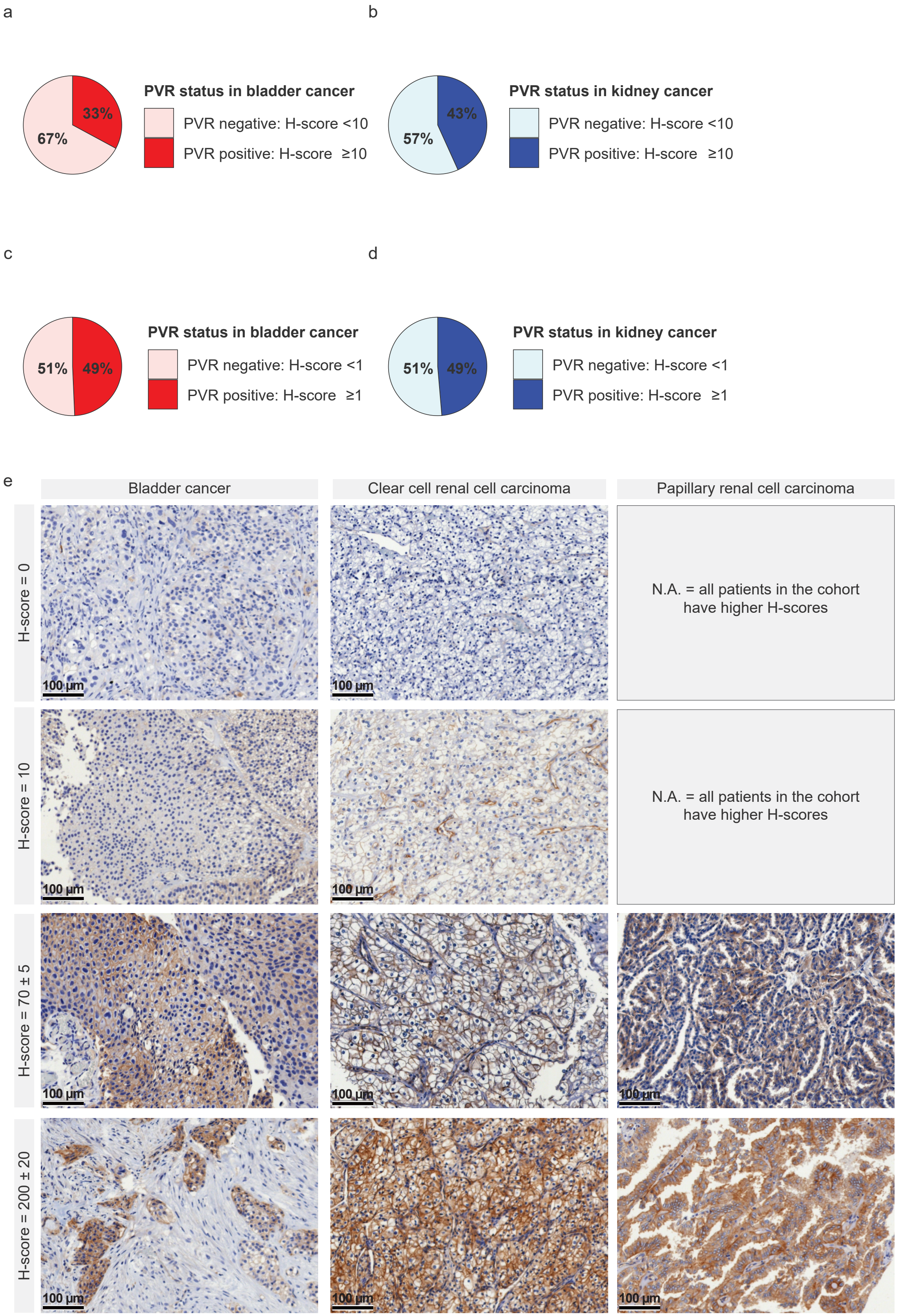

a
33%
67%
PVR status in bladder cancer
PVR negative: H-score <10
PVR positive: H-score ≥10
b
43%
57%
PVR status in kidney cancer
PVR negative: H-score <10
PVR positive: H-score ≥10
c
51%
49%
PVR status in bladder cancer
PVR negative: H-score <1
PVR positive: H-score ≥1
d
51%
49%
PVR status in kidney cancer
PVR negative: H-score <1
PVR positive: H-score ≥1
e
Bladder cancer
Clear cell renal cell carcinoma
Papillary renal cell carcinoma
H-score = 0
H-score = 10
H-score = 70 ± 5
H-score = 200 ± 20
N.A. = all patients in the cohort have higher H-scores
N.A. = all patients in the cohort have higher H-scores
100 µm

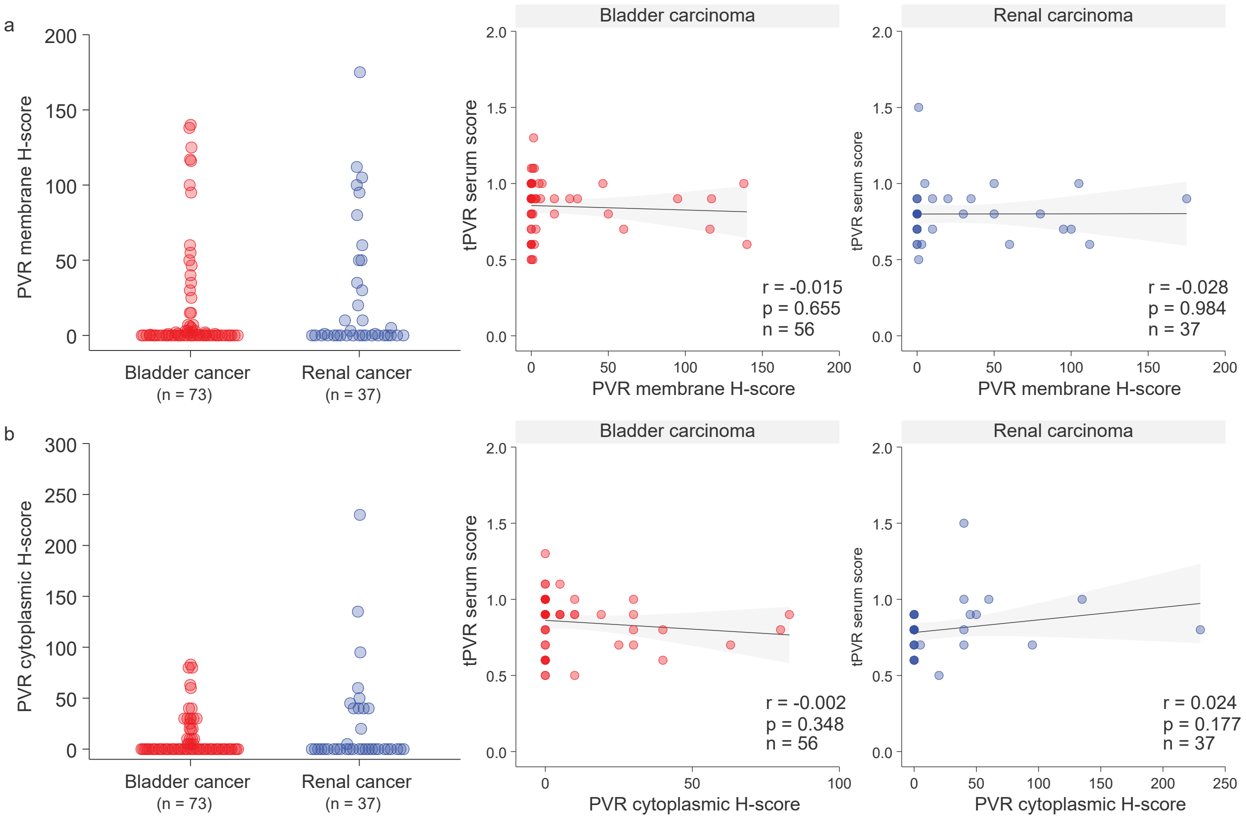
a
PVR membrane H-score
Bladder cancer
(n = 73)
Renal cancer
(n = 37)
Bladder carcinoma
tPVR serum score
PVR membrane H-score
r = -0.015
p = 0.655
n = 56
Renal carcinoma
tPVR serum score
PVR membrane H-score
r = -0.028
p = 0.984
n = 37
b
PVR cytoplasmic H-score
Bladder cancer
(n = 73)
Renal cancer
(n = 37)
Bladder carcinoma
tPVR serum score
PVR cytoplasmic H-score
r = -0.002
p = 0.348
n = 56
Renal carcinoma
tPVR serum score
PVR cytoplasmic H-score
r = 0.024
p = 0.177
n = 37

Supplementary Table 1. Bladder and renal cancer patient characteristics

Bladder cancer		Renal cancer	
All patients N= 73		All patients N= 37	
Characteristics	N	Characteristics	N
Median age of diagnosis			
<70	33	<67	17
≥70	40	≥67	20
Sex			
Male	60	Male	22
Female	13	Female	15
Type			
Papillary	62	Papillary	10
Non-papillary	9	Clear cell	25
Other[a]	2	Other[b]	2
Stage			
pTa, pT1	46	pT1, pT2	22
pT2, pT3, pT4	27	pT3, pT4	15
Grade			
Low grade	26	Low grade[c]	18
High grade	47	High grade[c]	18
Lymphovascular invasion (LVI)			
No LVI	48	No LVI	7
LVI	25	LVI	30
Metastatic disease[d]			
		Non-metastatic	25
		Metastatic	11

Note: [a]other bladder cancer type = squamous cell carcinoma, [b] other renal cancer type = chromophobe and oncocytic RCC, [c] renal low grade cancer = Fuhrman grade I, II, renal high grade cancer = Fuhrman grade III, IV, [d] Among the total cohort of 37 renal cancer patients, one individual could not be followed for the development of metastatic disease and bladder cancer patients were not followed for metastatic disease.

Supplementary table 2. Clear cell carcinoma patient characteristics

ccRCC	
All patients N=95	
Characteristics	N
Median age of diagnosis	
<69	48
≥69	47
Sex	
Male	70
Female	25
Stage	
pT1, pT2	62
pT3, pT4	33
Grade	
Low grade[c]	56
High grade[c]	39
Metastatic disease[d]	
Non-metastatic	57
Metastatic	37

Note: [a]Among the total cohort of 95 patients, one individual could not be followed for the development of metastatic disease.
